# Cyclic Peptides with Antifungal Properties Derived from Bacteria, Fungi, Plants, and Synthetic Sources

**DOI:** 10.3390/ph16060892

**Published:** 2023-06-18

**Authors:** Naiera M. Helmy, Keykavous Parang

**Affiliations:** 1 Center for Targeted Drug Delivery, Department of Biomedical and Pharmaceutical Sciences, Chapman University School of Pharmacy, Harry and Diane Rinker Health Science Campus, Irvine, CA 92618, USA; mohamedhelmy@chapman.edu; 2 Microbial Biotechnology Department, Biotechnology Research Institute, National Research Centre, Giza 3751134, Egypt

**Keywords:** antifungal, bacteria, cyanobacteria, cyclic peptides, fungi

## Abstract

Fungal infections remain a significant concern for human health. The emergence of microbial resistance, the improper use of antimicrobial drugs, and the need for fewer toxic antifungal treatments in immunocompromised patients have sparked substantial interest in antifungal research. Cyclic peptides, classified as antifungal peptides, have been in development as potential antifungal agents since 1948. In recent years, there has been growing attention from the scientific community to explore cyclic peptides as a promising strategy for combating antifungal infections caused by pathogenic fungi. The identification of antifungal cyclic peptides from various sources has been possible due to the widespread interest in peptide research in recent decades. It is increasingly important to evaluate narrow- to broad-spectrum antifungal activity and the mode of action of synthetic and natural cyclic peptides for both synthesized and extracted peptides. This short review aims to highlight some of the antifungal cyclic peptides isolated from bacteria, fungi, and plants. This brief review is not intended to present an exhaustive catalog of all known antifungal cyclic peptides but rather seeks to showcase selected cyclic peptides with antifungal properties that have been isolated from bacteria, fungi, plants, and synthetic sources. The addition of commercially available cyclic antifungal peptides serves to corroborate the notion that cyclic peptides can serve as a valuable source for the development of antifungal drugs. Additionally, this review discusses the potential future of utilizing combinations of antifungal peptides from different sources. The review underscores the need for the further exploration of the novel antifungal therapeutic applications of these abundant and diverse cyclic peptides.

## 1. Fungal Infections

In the past few years, there has been a notable surge in the focus on antifungal research. This increased attention can be attributed to several factors, including the improper utilization of antimicrobial drugs, the rise of resistance among microorganisms, and the escalating need for fewer cytotoxic antifungal agents to cater to immunocompromised patients. A few fungal infections are a significant concern for human health, especially in immunocompromised individuals.

*Candidiasis*, the most common opportunistic yeast infection, is caused by *Candida* species and other microorganisms, with *Candida albicans (C. albicans)* being the most prevalent. Excessive growth in the gastrointestinal, urinary, and respiratory tracts is not limited to immunocompromised patients and also occurs in healthy individuals, frequently resulting in nosocomial infections [1].

*Aspergillosis* is caused by *Aspergillus terreus*, *Aspergillus niger*, *Aspergillus flavus*, and *Aspergillus fumigatus*. Allergic syndromes to chronic pulmonary conditions and invasive infections are the consequences of the disease spectrum produced by *Aspergillus* species. Immunocompromised patients often face significant morbidity and mortality due to invasive *aspergillosis*, which is a leading cause of these adverse outcomes. The risk of *aspergillosis* was observed to increase in patients with post-influenza infection and after COVID-19 infection [2].

*Fusarium* species, comprising *Fusarium solani*, *Fusarium oxysporum*, *Fusarium moniliforme*, and *Fusarium verticilloides*, are the predominant filamentous pathogens found in high-risk patients. Among these species, *Fusarium solani*, originally a plant pathogen, is responsible for nearly 50% of fungal infections, which are also attributed to other opportunistic *Fusarium* species [3]. Thus, several antifungal agents have been developed to circumvent these pathogens and fungal infections. 

## 2. Antifungal Agents

Antifungal agents are comparable to antibiotics in their ability to inhibit growth, either reversibly (fungistatic versus bacteriostatic) or irreversibly (fungicidal versus bactericidal). These agents work by disrupting the essential reactions involved in fungal growth. This may include interference with the biosynthetic pathways responsible for building blocks, coenzymes, and macromolecules such as nucleic acids and proteins. Additionally, antifungal agents may target the maintenance and synthesis of cellular structures, such as the cell wall or cell membrane of pathogenic fungi. Understanding the mode of action of an antifungal agent involves discovering the process by which growth is inhibited and reactions are blocked [4]. Antifungal agents can target fungal sterols, nucleic acid, and β-glucan synthase as well as act through protein synthesis pathways; the inhibition of the *N*-myristylation of fungal proteins; the depolarization of mitochondrial membranes, the nucleus, or the endoplasmic reticulum; and the alteration of fungal cell membrane distribution [5].

**Figure 1:**
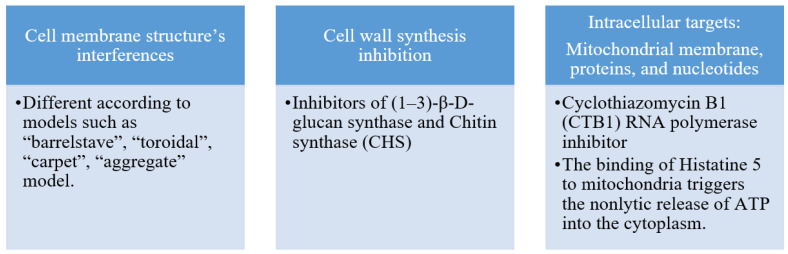
Mode of action of antifungal peptides [6].

Antifungal peptides primarily exert their mode of action on the cell membrane, cell wall, and other intracellular targets. Figure 1 summarizes the targets of antifungal peptides. The fungal cell wall is the first line of defense against the external environment and helps to regulate osmotic pressure. The inhibitory action of antifungal peptides on chitin synthase (CHS) and (1–3)-β-D-glucan synthase leads to the impairment of cell wall synthesis and disrupts normal cell morphology. This weakens the fungal cell′s ability to regulate osmotic pressure. Antifungal peptides can be selective in targeting the fungal cell membrane due to structural differences between plant and fungal cell membranes. This contributes to their effectiveness as plant antifungal agents. Antifungal peptides can also target intracellular substances, including mitochondrial membranes, proteins, and nucleotides such as DNA and RNA. The selective targeting and characterization of antifungal targets provides a useful tool for designing effective antifungal agents, as noted by Zhang et al. [6].

## 3. Cyclic Peptides Development as Antifungal Agents

Cyclic peptides are a distinct group of cyclic compounds composed of amino acids renowned for their distinctive architectures and extensive repertoire of biological activities. They arise from the linkage of multiple natural or unnatural amino acids in a precise sequence, creating a cyclic structure. These amino acids are intricately connected through amide or other chemically stable bonds, which can form between the *C*- and *N*-termini of the peptide sequence or between the peptide′s head and tail [7].

Compared to linear peptides, cyclic peptides offer several advantages. Firstly, under physiological conditions where spontaneous degradation may occur, peptide bonds are susceptible to proteolytic degradation. Converting peptides to their cyclic form enhances their in vivo stability when compared to their linear counterparts. This cyclization process was observed to extend the integrity of peptides with unstable motifs in their sequence. Thus, cyclic peptides demonstrated slower metabolism due to their high resistance to proteases, resulting in a longer reservoir effect compared to linear peptides [8].

Moreover, although the conformational flexibility of macrocyclic structures is limited, it brings about a reduction in the entropy value of drug target binding. Interestingly, this limitation enhances the binding stability of cyclic peptides, leading to higher affinity and recognition specificity between the cyclic peptides and the target protein [9].

Cyclic peptides possess unique characteristics that make them suitable as antimicrobial agents, including high potency, low toxicity, and specificity. This review primarily emphasizes the identification of cyclic peptides derived from various sources and the preliminary evaluation of their biological effects, along with future prospects and potential applications. This review specifically highlights the potential of cyclic peptides from various sources, such as bacteria, fungi, plants, and synthesis, as antifungal agents. This concise review does not intend to provide an exhaustive compilation of all known antifungal cyclic peptides. Instead, its purpose is to present diverse examples of cyclic peptides with antifungal properties sourced from various origins. Our aim is to offer the scientific community insights into the potential applications of these compounds as antifungal agents. Moreover, the inclusion of commercially available cyclic antifungal peptides strengthens the notion that cyclic peptides can serve as a valuable resource for the development of antifungal drugs.

**Figure 2:**
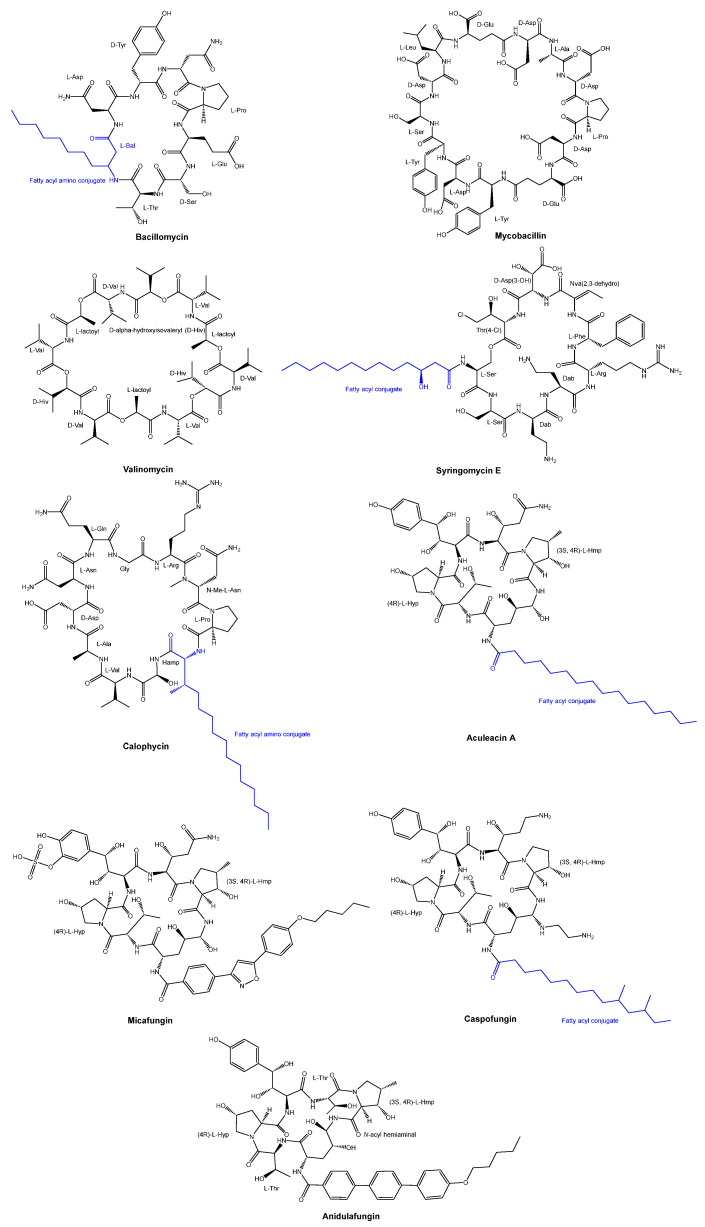
Chemical structures of Valinomycin, Syringomycin E, calophycin, Aculeacin A, and three antifungal cyclic peptide drugs (Micafungin, Caspofungin, and Anidulafungin) approved for clinical use in the last two decades.

The antifungal peptide Bacillomycin was obtained from *Bacillus subtilis* through isolation in 1948 [10]. Since 1960, there has been ongoing development of antifungal peptides. Among these, Mycobacillin was discovered in 1963 from the biosynthesis in *Bacillus subtilis* [11]. Valinomycin was discovered in the early 1960s as a cyclodepsipeptide from a species of *Streptomyces* [12], while Syringomycin E, a lipodepsipeptide, was produced from *Pseudomonas syringae pv. Syringae* [13,14]. Another example is the antifungal cyclic decapeptide called calophycin [15], which was isolated from the terrestrial blue-green alga *Calothrix fusca*. Furthermore, Aculeacin A, a cyclopeptide that incorporates a long-chain fatty acid, was obtained from the mycelial cake of *Aspergillus aculeatus M-4214* [16,17,18]. The chemical structures of Valinomycin, Syringomycin E, calophycin, and Aculeacin A are illustrated in Figure 2. 

In the last two decades, three cyclic hexapeptides with antifungal properties, caspofungin, micafungin, and anidulafungin, have received approval for clinical use (Figure 2). All three antifungal agents demonstrated clinical efficacy in treating invasive *candidiasis* and various types of systemic fungal infections [19]. These three agents target the 1,3-β-glucan synthase involved in the cell wall glucan synthesis. 

Caspofungin received approval in 2001, while micafungin was granted approval in 2005. These compounds possess a common feature in their structure, which is a shared peptide core comprising six amino acids. Among these amino acids, two are threonine or threonine derivatives, while the remaining two are proline derivatives [20]. 

Micafungin, a semi-synthetic analog of the natural cyclic hexapeptide FR901379 [21], demonstrates remarkable antifungal potency against *Aspergillus* spp. and *Candida* spp. [22]. It is characterized by a linear pharmacokinetic profile and a significantly lower occurrence of adverse reactions compared to amphotericin B. These attributes make micafungin highly valuable for effectively treating deep-seated fungal infections. In both adult and pediatric patients, micafungin exhibits absorption pharmacokinetics that are linear and dose-proportional across a wide dosage range [23]. However, micafungin differentiates itself from the other two compounds by having low oral bioavailability and being exclusively administered via parenteral routes. It possesses a half-life of approximately 15 h. The majority of micafungin undergoes metabolism and is excreted through feces as a result of enzymatic modifications in its side chains [24].

Caspofungin, approved for the treatment of yeast and fungal infections under specific conditions in 2001 [25], is a semi-synthetic analog of pneumocandin B0. Pneumocandin B0 is a lipophilic cyclic peptide initially derived from the fungus *Glarea lozoyensis* in 1985 [26]. It exhibits a half-life of around nine days and is primarily metabolized through metabolic hydrolysis and *N*-acetylation, causing the cyclic ring to open. Micafungin, in contrast, is a semi-synthetic derivative of a natural cyclic hexapeptide FR901379. The enzymatic and chemical deacylation processes of the *N*-terminal palmitoyl group of FR901379 result in the formation of the optimal *N*-acyl isoxazole analog [27].

Anidulafungin is a natural fermentation product of *Aspergillus oryzae* and is derived from echinocandin B through diacylation to eliminate the linoleoyl side chain, followed by reacylation with the lipophilic terphenylacyl chain after three additional steps [20,28]. Anidulafungin exhibits clinical effectiveness in the inhibition of invasive candidiasis by employing a mechanism of noncompetitive inhibition on β-(1,3)-glucan synthase [29]. Furthermore, anidulafungin demonstrates a low oral bioavailability ranging from 2% to 7%, necessitating non-intestinal administration [30]. With a half-life of 27 h, anidulafungin primarily undergoes metabolic conversion into linear peptides through open-loop hydrolysis.

The sources of cyclic antifungal peptides vary and can include (a) cyanobacteria and other bacteria, (b) fungi, (c) plants, and (d) synthetic methods. The following sections provide examples of cyclic peptides from each of these sources.

### 3.1. Cyclic Peptides Isolated from Cyanobacteria 

Cyanobacteria are a type of blue-green algae known to produce biologically active cyclic peptides and depsipeptides, including microcystins and lyngbyatoxins, which have a structure similar to that found in marine invertebrates. 

Cyclic lipopeptides derived from cyanobacteria have shown remarkable antifungal activity against a range of human and plant fungal pathogens, alongside their antibiotic properties. The amphipathic structure of these peptides consists of a polar peptide cycle and a hydrophobic fatty acid side chain. Given their potential as new antifungal agents, there is growing interest in exploring the novel biotechnological and therapeutic applications of these abundant and diverse cyclic peptides derived from cyanobacteria.

**Figure 3:**
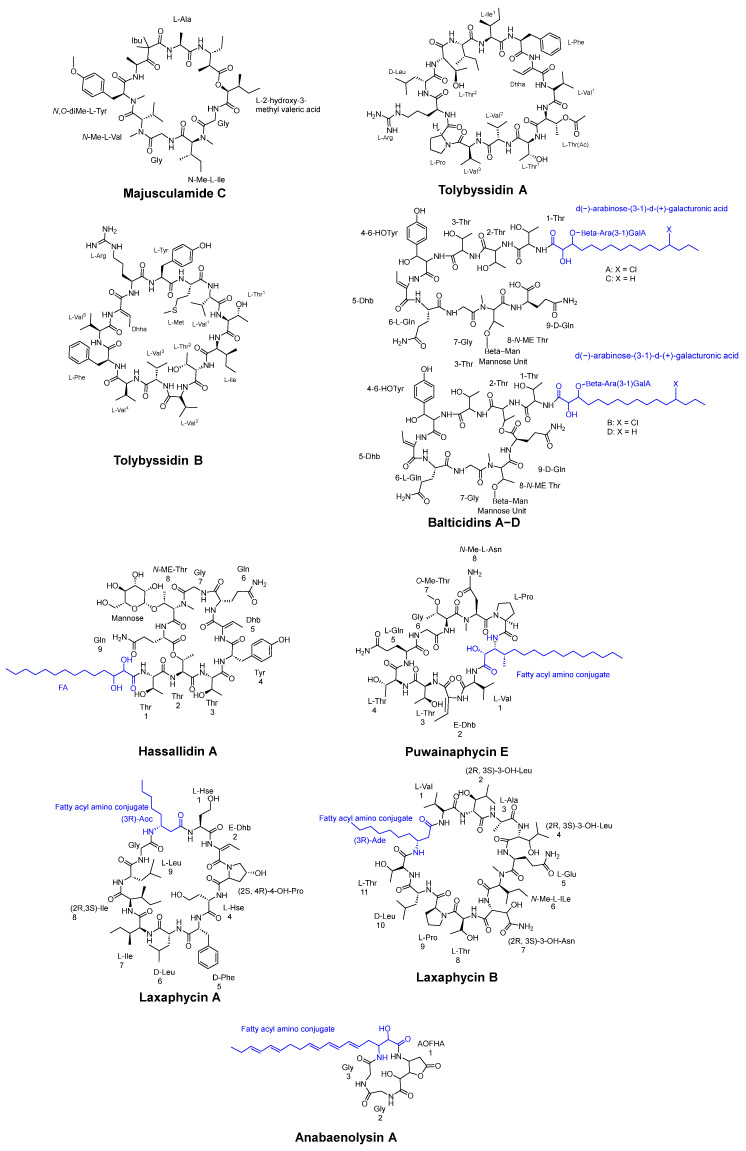
Some cyclic peptides isolated from Cyanobacteria include Tolybyssidins A and B, Majusculamide C, Anabaenolysin A, Balticidins A-D, Puwainaphycin E, laxaphycin A and B, and Hassallidin A.

One example of these compounds is Majusculamide C (Figure 3), a potent fungicidal agent extracted from *Lungbya majuscula*. This microfilament-depolymerizing agent was shown to be effective against diseases in domestic plants and agricultural crops [31].

*Tolypothrix byssoidea* (EAWAG 195), a cyanobacterium, was found to produce two cyclic tridecapeptides known as tolybyssidins A and B (Figure 3). Peptides A and B exhibit antifungal activity against *C. albicans* at concentrations of 32 µg/mL and 64 µg/mL, respectively, and contain the non-natural amino acid dehydrohomoalanine (Dhha), along with proteinogenic amino acids, some of which have a D- or L-configuration in their structure [32]. 

The cyanobacterium *Anabaena cylindrica* strain Bio33 was obtained from a water sample collected near Rügen Island, Germany, in the Baltic Sea. This isolate was found to contain four antifungal lipopeptides known as Balticidins A−D, which were characterized and extracted (Figure 3). These peptides were shown to exhibit antifungal activity by generating inhibition zones from 21 to 32 mm using the agar diffusion method against various strains including *Candida maltosa*, *Candida krusei*, *C. albicans*, *Microsporum gypseum*, *Microsporum canis*, *Mucor* sp., and *Aspergillus fumigatus* [33].

Cyclic antifungal lipopeptides derived from cyanobacteria can be also categorized into four main structural classes: hassallidins, puwainaphycins, laxaphycins, and anabaenolysins (Figure 3). Hassallidins, produced by various cyanobacteria, are cyclic glycosylated lipopeptides consisting of a fatty acid chain, a peptide ring comprising eight amino acids, an exocyclic amino acid, and 1–3 sugar moieties. Puwainaphycins are amphipathic cyclic lipopeptides characterized by a β-amino fatty acid and a nine-membered peptide ring. Laxaphycins form a diverse group of cyclic lipopeptides, typically containing a rare β-amino fatty acid with a short linear chain of 8 or 10 carbons. Anabaenolysins, another class of cyclic lipopeptides, feature an uncommon unsaturated β-amino fatty acid with a conjugated triene structure and a four-membered peptide ring [34].

**Figure 4:**
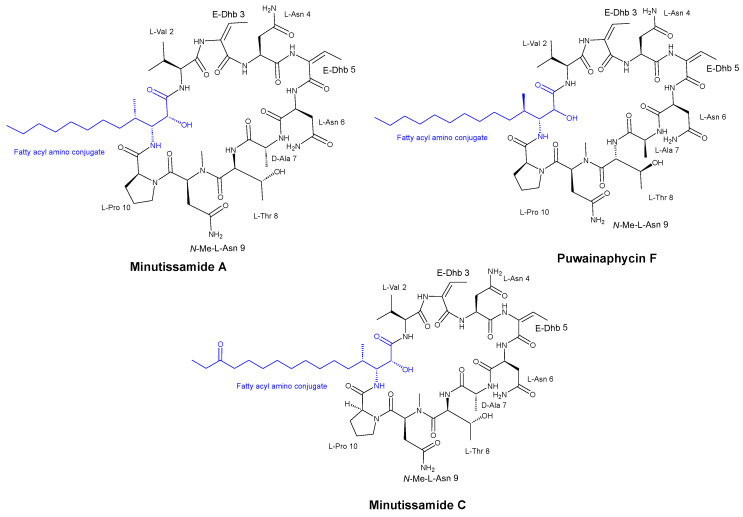
Structures of isolated compounds MIN A, PUW F, and MIN C.

Hájek et al. [35] discovered that the cyclic lipopeptides from cyanobacteria, known as the puwainaphycin/minutissamide (PUW/MIN) family (Figure 4) and a number of semisynthetic analogs, exhibited antifungal activity against both *Alternaria alternata* (a plant pathogen) and *Aspergillus fumigatus* (a human pathogen) with MIC values of 37 μM and 0.6 μM, respectively, suggesting a correlation between their antifungal activities. Moreover, the researchers observed an enhancement of the antifungal properties of these peptides through the generation of semi-synthetic lipopeptides. 

### 3.2. Cyclic Peptides Isolated from Other Bacteria

**Figure 5:**
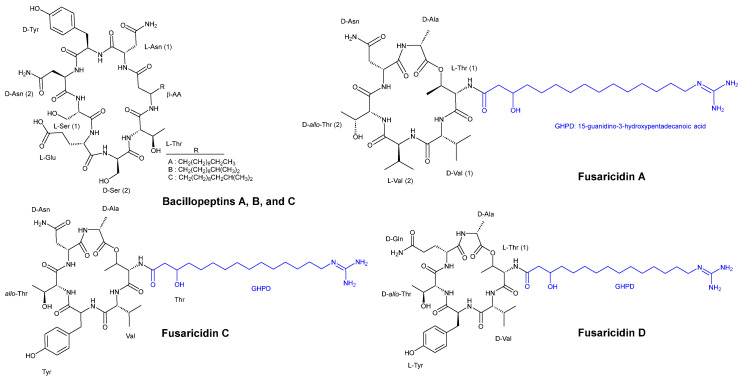
Chemical structures of bacillopeptins A, B, and C and fusaricidins A, C, and D.

Kaneda and Kajimura [36] obtained four strains of bacteria isolated from the rhizosphere of garlic with basal root due to the plant pathogenic *Fusarium oxysporum*. Among these strains, *Bacillus subtilis FR-2* was discovered to produce three cyclic lipopeptides, namely, bacillopeptins A, B, and C (Figure 5), each incorporating a long-chain beta-amino acid. Compounds of bacillopeptins A, B, and C showed MIC values of >100 µg/mL, >100 µg/mL, and 50 µg/mL, respectively, against *C. albicans IFO 1594*. For *Saccharomyces cerevisiae HUT 7099*, the MIC values were >100 µg/mL, >100 µg/mL, and 25 µg/mL for compounds A, B, and C, respectively. The MIC values for the two strains *Fusarium oxysporum HF 8801* (pathogenic to garlic) and *F. oxysporum HF 8835* (nonpathogenic to garlic) were >100 µg/mL and >100 µg/mL for compounds A and B, respectively, while compound C exhibited a MIC value of 25 µg/mL for the pathogenic strain and 12.5 µg/mL for the nonpathogenic strain. Compounds A and B demonstrated similar activity against *Aspergillus niger HUT 2016*, with a MIC value of >100 µg/mL, whereas compound C showed a MIC value of 6.25 µg/mL. Compounds A, B, and C showed MIC values of >100 µg/mL, >100 µg/mL, and 12.5 µg/mL, respectively, against *A. oryzae IFO 4214*. The same MIC values were also observed against *Penicillium thomii*. 

*Bacillus polymyxa KT-8*, another strain, was identified as a producer of the highly effective antibiotic and antifungal cyclic hexadepsipeptides, namely, fusaricidins A, B, C, and D (Figure 5). These compounds all contain 15-guanidino-3-hydroxypentadecanoic acid as a side chain. Fusaricidins exhibited higher antifungal activity compared to bacillopeptins. The antifungal activity of fusaricidins A, B, and a mixture of C and D against *Saccharomyces cerevisiae HUT 7099* showed MIC values of >100 µg/mL, 12.5 µg/mL, and >100 µg/mL, respectively. The lowest MIC value of 1.56 µg/mL was observed against *Fusarium oxysporum HF 8801* (pathogenic to garlic) and *F. oxysporum HF 8835* (nonpathogenic to garlic). Fusaricidins A, B, and a mixture of C and D exhibited identical MIC values of 3.12 µg/mL against *Aspergillus niger HUT 2016*, *A. oryzae IFO 4214*, and *Penicillium thomii*. 

**Figure 6:**
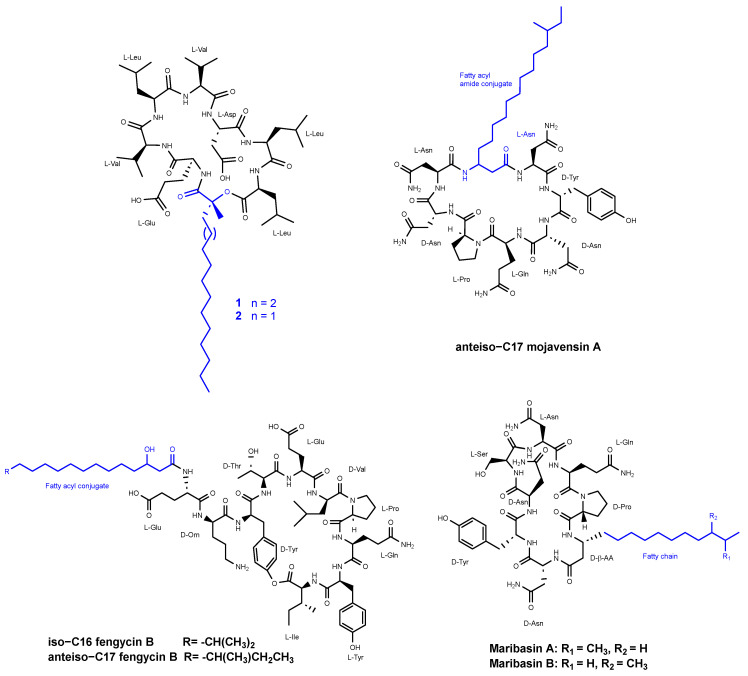
Structures of compounds **1** and **2** from the surfactins family, anteiso-C17 mojavensin A (**1**), iso-C16 fengycin B, and anteiso-C17 fengycin B, and two cyclic lipopeptides, maribasins A and B.

Romano et al. [37] reported the discovery of two new cyclic lipopeptides (designated as **1** and **2**) (Figure 6) belonging to the surfactins family, isolated from *Bacillus amyloliquefaciens* strain BO5A. The lipopeptides are composed of a heptapeptide chain with the amino acid sequence Glu-Val-Leu-Val-Asp-Leu-Leu and are *N*-acylated at their *N*-terminal end by (R)-3-hydroxy fatty acids with linear alkyl chains of 16:0 and 15:0 for compounds **1** and **2**, respectively, through the formation of a 25-membered lactone ring between the 3-hydroxyl group of the fatty acid and the carboxylic group of the *C*-terminal amino acid. Compound **2** displayed inhibitory antifungal activity against *Aspergillus niger*, *Fusarium oxysporum*, *Penicillium italicum*, and *Trichoderma harzianum* at three tested concentrations of 10, 50, and 100 ppm. Compound **1** demonstrated inhibition of 59% and 36% against *F. oxysporum* and *A. niger*, respectively. When a mixture of compounds **1** and **2** was evaluated, there was not any significant change in the antifungal activity. 

Ma et al. [38] reported the isolation of three lipopeptides from the fermentation broth of *Bacillus mojavensis B0621A* that exhibited dose-dependent antifungal activity against a broad range of phytopathogens. Among these lipopeptides, there is one called anteiso-C17 mojavensin A (Figure 6), which belongs to the iturinic lipopeptide family. The antifungal activity of anteiso-C17 mojavensin A was detected when the concentration was over 2 mg/mL for *Valsa mali, Fusarium oxysporum f. sp. cucumerinum,* and *Fusarium verticillioides*. This compound possesses a distinctive peptide backbone composed of L-Asn_1_, D-Tyr_2_, D-Asn_3_, L-Gln_4_, L-Pro_5_, D-Asn_6_, and L-Asn_7_. Additionally, it features an anteiso-type saturated β-fatty acid side chain. 

The other two compounds, tentatively identified as iso-C16 fengycin B and anteiso-C17 fengycin B (Figure 6), were also assessed for their antifungal activities. The compounds resulted in inhibition zones ranging from 5.17 ± 0.19 to 11.88 ± 0.47 mm when tested at concentrations of 2 mg/mL and 3 mg/mL. These inhibitory effects were observed against various fungal strains, including *Valsa mali*, *Fusarium oxysporum f. sp. cucumerinum*, *Fusarium oxysporum f. sp. vasinfectum*, *Fusarium oxysporum f.* sp., *vasinfectum*, *SF2*, *Fusarium solani SF 130*, *Botryosphaeria berengriana f. sp. piricola*, *Botrytis cicrea*, *Rhizoctonia solani J. G. Kühn, Fusarium solani, Rhizoctonia solani, Valsa ceratosperma, Fusarium oxysporum f. sp.*, *Cucumis melo* L., *Fusarium graminearum*, *Bipolaris maydis*, *Colletotrichum orbiculare*, *Fusarium verticillioides*, and *Fusarium verticillioid*.

Zhang et al. [39] identified two cyclic lipopeptides, maribasins A and B (Figure 6), derived from the marine microorganism *Bacillus marinus B-9987*. This microorganism was isolated from *Suaeda salsa*, a plant species found along the Bohai coastline in the People′s Republic of China. The structures of both compounds were determined to be cyclo (D-Pro-L-Gln-L-Asn-L-Ser-D-Asn^1^-D-Tyr-D-Asn^2^-D-β-aminoisopentadecanoic acid) and cyclo (D-Pro-L-Gln-L-Asn-L-Ser-D-Asn-D-Tyr-D-Asn-D-β-aminoanteisopentadecanoic acid). 

Maribasins A and B exhibited broad-spectrum activities, demonstrating MIC values that ranged from 25 to 200 µg/mL against various fungi, including *Alternaria solani*, *Fusarium oxysporum*, *Verticillium alboatrum*, *F. graminearum*, *Sclerotium* sp., *Penicillium* sp., *Rhizoctonia solani*, and *Colletotrichum* sp.

**Figure 7:**
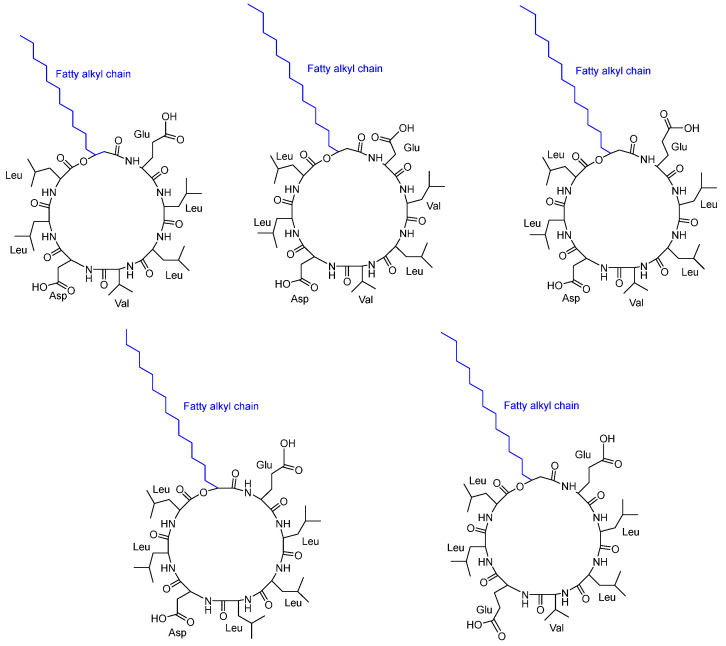
Structure of five cyclic lipopeptides (CLPS) obtained from *Neobacillus drentensis* AKLSR2. Stereochemistry of these compounds was not reported.

Routhu et al. [40] discovered that the biofilm exopolymeric substances (EPS) surrounding marine microbes possess antifungal activity against *C. albicans* pathogenic strains (susceptible) and one azole-resistant *Candida* strain. They characterized five cyclic peptides CLPs (Figure 7) isolated from the marine microbe *Neobacillus drentensis.* The five CLP isoforms were recognized as new peptides with variants in the amino acid sequence and fatty acid chain. All the tested *Candida* sp. was inhibited by the least concentration of CLPs, depending on the variety of the strain. The MIC value was found to be 7.8 µg/mL against two strains of *C. albicans*. The inhibition of *C. albicans*’s growth was characterized by preventing biofilm formation and disrupting the branching of filamentous hyphae. The cyclic peptides were considered to play a role in blocking the G1–S transition and inducing apoptotic cell death. The stereochemistry of the amino acids was not reported.

**Figure 8:**
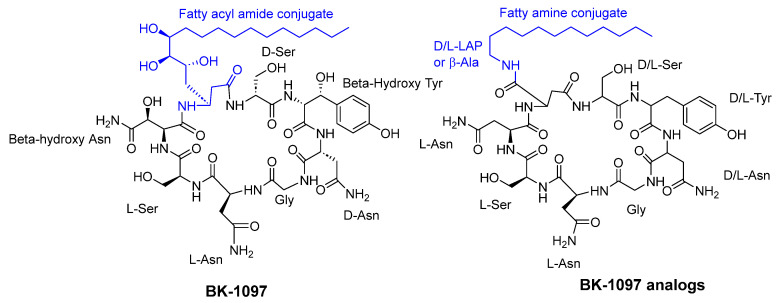
BK-1097 and BK-1097 analogs.

Konno et al. [41] reported that cyclic octapeptides derived from burkholdines exhibit antifungal activity against *Saccharomyces cerevisiae*, *Aspergillus oryzae*, and *Candida viswanathii*. The most potent analogs of burkholdines showed MIC values ranging from 25 to 50 µg/mL. The antifungal activity of BK-1097 was notably affected by both the lipid side chain and the stereochemistry of each amino acid present (see Figure 8). 

Troskie and colleagues [42] reported the production of cationic cyclodecapeptides by *Bacillus aneurinolyticus*, including Tyrocidine A (TrcA), Tyrocidine B (TrcB), Tyrocidine C (TrcC), Phenycidine A (PhcA), Tryptocidine C (TpcC), and Gramicidin S [42]. The sequences of these peptides are as follows: cyclo-(VOLfPFfNQY), cyclo-(VOLfPWfNQY), cyclo-(VOLfPWwNQY), cyclo-(VOLfPFfNQF), cyclo-(VOLfPWwNQW), and cyclo-(VOLfPVOLfP), respectively. The tyrocidines were found to exhibit antifungal activity against *C. albicans* and effectively inhibit biofilm formation in vitro. A mixture of Tyrocidines (TrcA, TrcB, TrcC, TpcC, and PhcA) displayed potent antifungal activity against planktonic *C. albicans*, with a MIC value of 6.25 µg/mL. Purified tyrocidines, TpcC, and the analogous peptide GS showed MIC values of 6.25 µM, while a higher MIC of 12.5 µM was detected with PhcA. The inhibitory effect of tyrocidines on mature *C. albicans* biofilm cells is attributed to their disruptive impact on the membrane integrity [43].

Troskie and colleagues [44] evaluated the antifungal activity of the cationic cyclodecapeptides produced by *Bacillus aneurinolyticus* against *Aspergillus fumigatis ATCC 204305*, *Fusarium solani STEU 6188*, *Fusarium oxysporum ATCC 10913*, *Fusarium verticilliodes CKJ1730*, *Botrytis cinerea CKJ1731*, *Cylindrocarpon liriodendri STEU 6170*, *Penicillium glabrum CKJ1732*, *Talaromyces ramulosus CKJ1735*, *Talaromyces mineoluteus CKJ1736*, *Penicillium expansum CKJ1733*, and *Penicillium digitatum CKJ1734.* The tyrocidine peptide complex (Trc mixture) and purified tyrocidines exhibited antifungal activity, with MIC values of below 13 mg/mL (~10 µM). The antifungal mechanism involved the inhibition of germination and the process of hyphal hyper-branching. The inhibitory impact of tyrocidines on the membrane integrity was found to be responsible for their inhibitory effect. 

Rautenbach and colleagues [45] conducted a study on tyrocidines and their analogs, which are cyclic decapeptides produced by *Brevibacillus parabrevis*. These peptides shared a conserved sequence of cyclo(D-Phe^1^-Pro^2^-X^3^-X^4^-Asn^5^-Gln^6^-X^7^-Val^8^-X^9^-Leu^10^), where X_3_, X_4_, X_7_, and X_9_ represent variable amino acid residues. The aromatic dipeptide unit can be either Trp^3,4^ or Phe^3,4^, and Lys^9^/Orn^9^ serves as the cationic residue. At position 7, the peptides contain either Tyr (tyrocidines), Trp (tryptocidines), or Phe (phenicidines). The researchers observed that replacing the Tyr residue at position 7 with Phe or Trp enhanced the antifungal activity of the peptides. The most active peptides displayed IC_50_ (µg/mL) values of 1.1, 2.6, and 2.6 against *Botrytis cinerea CKJ1731*, *Fusarium solani STEU 6188*, and *Aspergillus fumigatis ATCC 204,305*, respectively.

Overall, cyclic peptides isolated from cyanobacteria and other bacteria have great potential as new antifungal agents and for other biotechnological and therapeutic applications. There is growing interest in exploring the novel properties and applications of these abundant and diverse cyclic peptides.

### 3.3. Cyclic Peptides Isolated from Fungi

In general, several studies reported the isolation of cyclic peptides from various fungal strains and their potential as antifungal agents. These findings suggest that cyclic peptides from fungi hold great promise as potential sources of novel antifungal agents.

**Figure 9:**
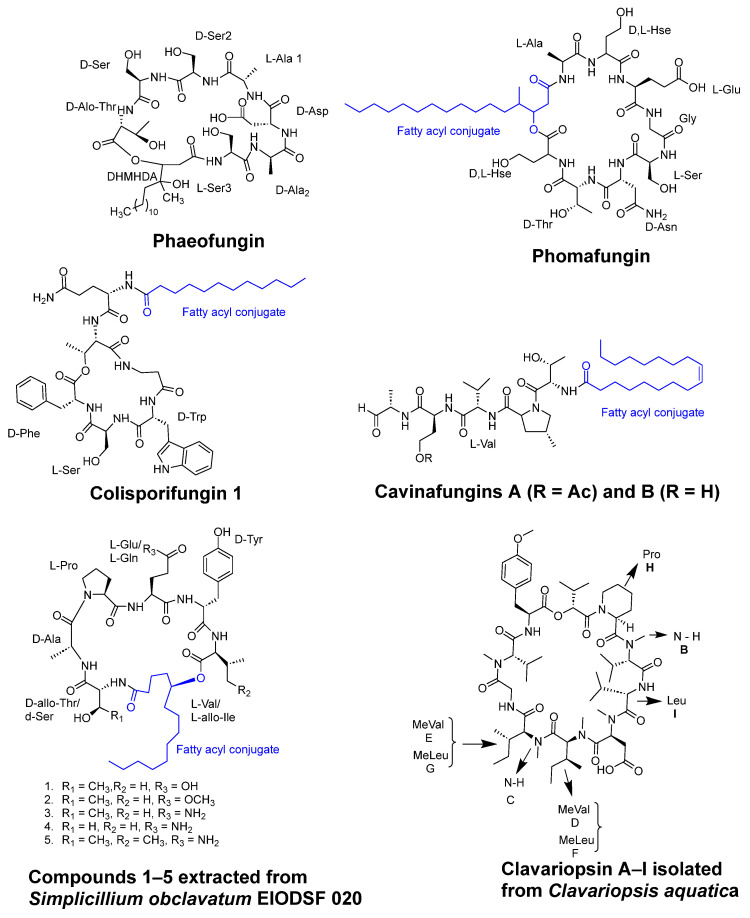
The structures of cyclic peptides isolated from fungi.

Using reverse genetics technology and the *C. albicans* fitness test (CaFT), the cyclic desipeptide phaeofungin (Figure 9) was isolated from *Phaeoshaeria* sp. This compound consists of a 25-membered cyclic depsipeptide made up of seven amino acids and a β,γ-dihydroxy-γ-methylhexadecanoic acid. In a similar approach, the CaFT profile of the phaeofungin-containing extract was found to overlap with that of phomafungin, a structurally different cyclic lipodepsipeptide also produced by the same organism [46]. 

Functionally, phaeofungin and phomafungin are distinct from each other. The potentiation of phomafungin′s antifungal activity was observed when combined with cyclosporin A, an inhibitor of the calcineurin pathway. In contrast, phaeofungin exhibited synergistic effects when combined with aureobasidin A 2, a sphingolipid biosynthesis inhibitor, and, to some extent, with caspofungin, a glucan synthase inhibitor. Interestingly, phaeofungin induced ATP release in wild-type strains of *C. albicans*, while phomafungin did not exhibit this effect. It displayed antifungal activity against *Aspergillus fumigatus* (MIC 8–16 µg/mL), *C. albicans* (MIC 16–32 µg/mL), and *Trichophyton mentagrophytes* (MIC 4 µg/mL). Notably, the inactivity of the linear peptide reflected the importance of the macrocyclic depsipeptide ring for target engagement and activity as an antifungal compound [46]. 

Colisporifungin, a cyclic desilipopeptide with structural similarities to the linear peptides aselacins and cavinafungins A and B (as depicted in Figure 9), was isolated from liquid culture broths of the *Colispora cavincola* fungus. In experimental studies, colisporifungin exhibited a remarkable enhancement of the antifungal growth activity of caspofungin against *A. fumigatus* (MIC 8 µg/mL) and, to a lesser extent, *C. albicans* (MIC 0.5–4 µg/mL) [47].

Liang et al. [48] investigated the structure–bioactivity relationship of cyclic peptides derived from the deep-sea fungus strain *Simplicillium obclavatum EIODSF 020*. The study primarily examined the influence of the lactone linkage and the substituent group of the fatty acid chain fragment on the bioactivity of these cyclic peptides. These cyclic peptides included simplicilliumtides J–M, along with verlamelins A and B analogs. Out of these compounds, three peptides demonstrated antifungal activity against *Aspergillus versicolor* (IC_50_ = 14 µM) and *Curvularia australiensis* (IC_50_ = 16.7 µM).

In another study, the deep-sea-derived fungal strain *Simplicillium obclavatum* EIODSF 020 yielded two novel cyclopeptides, namely, simplicilliumtides N and O (**1** and **2**), along with three previously identified analogs, verlamelins A and B and simplicilliumtide J (**3**–**5**) (as depicted in Figure 9). These peptides exhibited notable antifungal activity against two phytopathogenic fungal strains, *Colletotricum asianum* and *Alternaria solani* (MIC= 0.195–6.25 µg/disc) [49].

A variety of peptides were isolated from the marine-gorgonian-associated fungus *Aspergillus sp. SCSIO 41,501* (Trichocomaceae), including three cyclic lipopeptides (Maribasins C-E), four linear peptides (Aspergillipeptides H-K), and three previously identified cyclic lipopeptides (Maribasins A-B and Marihysin A). Notably, Maribasins C-E and Maribasins A-B exhibited remarkable antifungal activity against five phytopathogenic fungal strains: *F. oxysporum, C. australiensis*, *P. oryzae*, *C. gloeosporioiles*, and *A. solani* (MIC 3.12–50 µg/disc). The structure–bioactivity relationship revealed that the antifungal activity of these cyclic lipopeptides could be significantly affected by the β-amino fatty acid chain [50].

*Clavariopsis aquatica*, an aquatic hyphomycete, was found to contain seven cyclic depsipeptides, clavariopsins C-I, and two previously identified compounds, clavariopsins A and B (Figure 9), all of which possess antifungal properties against six plant pathogenic fungi: *Magnaporthe oryzae*, *Botrytis cinerea*, *Fusarium oxysporum*, *Colletotrichum orbiculare*, *Aspergillus niger*, and *Alternaria alternata*. These peptides are composed of nine amino acids and one α-hydroxy acid and share the same structure. The antifungal activity showed values of minimum inhibitory dose ranging from 0.01 to 10 µg/disc [51].

It was reported that *Aureobasidium pullulans R106* produces aureobasidins, which possess antibiotic and antifungal properties. A member of this group is Aureobasidin A, which is a cyclic depsipeptide composed of eight alpha-amino acid units and one hydroxy acid unit. Studies showed that in addition to having a broad spectrum of activity, Aureobasidin A is more effective against murine *candidiasis* than the aculeacin/echinocandins family. The antifungal activity against *C. albicans*, *C. kefyr*, *C. glabrata*, and *Cryptococcus neoformans* showed MIC values ranging from 0.05 to 25 µg/mL [52].

### 3.4. Cyclic Peptides Isolated from Plants

Antifungal activity was observed in the crude ethanolic extracts derived from cultured Anabaena laxa, a blue-green alga, which was attributed to the presence of Laxaphycins. Antifungal activity against *Aspergillus oryzae*, *C. albicans,* *Penicillium notatum*, *Saccharomyces cerevisiae*, and *Trichophyton mentagrophytes* was indicated by the inhibition zone ranging from 8 to 26 mm in an agar diffusion. These cyclic peptides displayed an atypical biological synergism during the screening process for antifungal activity, as reported by Frankmölle et al. in [53].

**Figure 10:**
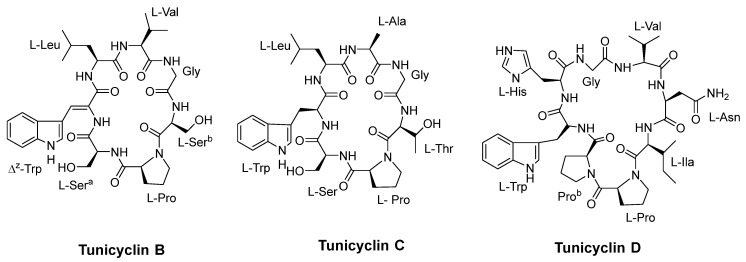
Structure of Tunicyclins B–D.

Tunicyclins B-D (Figure 10), cyclic peptides extracted from the root of Psammosilene tunicoides, showed antifungal activity against various strains. Among these, tunicyclin D demonstrated a broad spectrum of antifungal activity against various strains. It showed a MIC_80_ value of 4 µg/mL against *C. albicans (SC5314)* and slightly observed activity against *Candida tropicalis*. Tunicyclin D displayed the same MIC_80_ value of 1 µg/mL against both *Candida parapsilosis* and *Cryptococcus neoformans BLS108*, as reported by Tian et al. [54].

In addition to cyclic peptides, conformationally constrained peptides were shown to exhibit antifungal activity. The identification of small, basic, cysteine-rich peptides known as plant defensins has proven significant in the search for antifungal agents. These conformationally constrained peptides demonstrate potent antifungal activity against various fungal and yeast species at micromolar concentrations. Plant defensins exhibit selective activity and are non-phytotoxic, owing to the structural dissimilarities between the membrane components of plant and fungal cells. The antifungal activity of defensins in plants is a result of their interaction with specific lipid components in the plasma membrane of fungi, as reported by Thevissen et al. [55].

Orbitides and cyclotides are cyclic peptides that are derived from plants. They possess a distinct structural arrangement and sequence. Cyclotides, in contrast to orbitides, do not contain a disulfide bond and have a higher proportion of hydrophobic residues. Integerrimides A and B are orbitides isolated from the latex of *Jatropha integerrima* Jacq. In an antifungal assay, both compounds A and B exhibited no activity at concentrations of 64.1 µM and 65.3 µM [56,57].

Cyclotides, in contrast, possess a peptide backbone that forms a circular structure, typically composed of around 30 residues. They are characterized by the presence of six conserved cysteine residues, which form three disulfide bonds, creating a unique cyclic cystine knot motif [58]. Strömstedt et al. [59] evaluated the fungicidal activity of cycloviolacin O2, cycloviolacin O3, and cycloviolacin O19 against the fungi *C. albicans*. These cyclotides demonstrated a fungicidal activity of >99% with a Minimum Fungicidal Concentration (MFC) of 10 μM.

Thus, cyclic peptides and conformationally constrained peptides, such as defensins, that are isolated from plants have shown potent antifungal activity against various fungal and yeast species, at micromolar concentrations, owing to their interaction with specific lipid components in the plasma membrane of fungi. Furthermore, plant defensins exhibit selective activity and are non-phytotoxic, making them a promising avenue for antifungal drug development.

### 3.5. Synthetic Cyclic Peptides with Antifungal Activity

**Figure 11:**
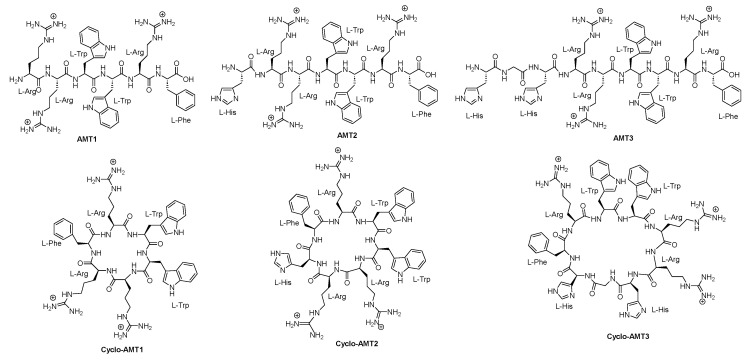
Structures of AMT1, AMT2, AMT3, cyclo-AMT1, cyclo-AMT2, and cyclo-AMT3.

Grimaldi and colleagues [60] synthesized and characterized six peptides, namely, AMT1, AMT2, AMT3, cyclo-AMT1, cyclo-AMT2, and cyclo-AMT3 (Figure 11), to assess their antifungal properties. The antifungal activity of these compounds was assessed against *Candida* species, including *C. albicans*, *C. glabrata*, and *C. tropicalis*. The linear peptides exhibited MIC values ranging from 64 to >512 µg/mL, while the cyclic peptides showed MIC values ranging from 32 to 512 µg/mL Generally, the cyclic peptides displayed greater activity compared to their linear counterparts. Moreover, all the tested compounds displayed potent antifungal activity against *C. neoformans* ATCC 52,817 and *C. neoformans* H99, with MIC values ranging from 4 to 16 µg/mL.

**Figure 12:**
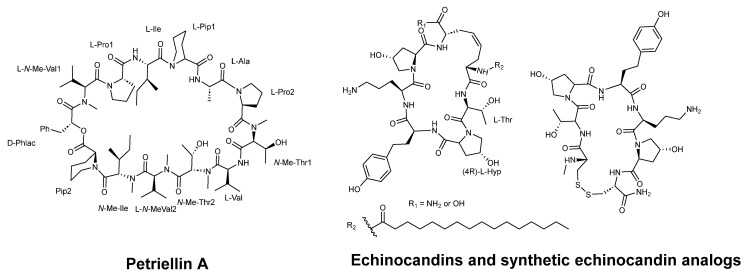
Structures of Petriellin A, echinocandins, and synthetic echinocandin analogs (adopted from [61,62]).

Sleebs et al. [61] described the synthesis of petriellin A (Figure 12), which was originally extracted from the organic extracts of *Petriella sordida UAMH 7493*, an antagonistic coprophilous fungus. This cyclic depsipeptide is composed of 1 R-D-hydroxycarboxylic acid and 12 L-amino acids, 5 of which are *N*-methylated. The study demonstrated that petriellin A displayed strong antifungal activity against *Ascobolus furfuraceous (NRRL 6460)* and *Sordaria fimicola (NRRL 6459)*.

Kurokawa and Ohfune [63] synthesized oligopeptides echinocandins C and D with fungicidal activity. Echinocandins, as macrocyclic peptides, inhibit the β-(1,3)-D-glucan synthase complex, a mechanism that provides selective activity, which is absent in mammalian cells. The antifungal activity of these peptides (see Figure 12) against *C. albicans CBS 9975* was evaluated, showing MIC values ranging from 0.025 to >100 µg/mL [62].

The glycosylic cyclic peptide antifungal drug caspofungin derivatives prepared from conjugation with β-d-glucopyranose, β-d-gal-actopyranose, β-d-xylopyranose, β-maltose, β-l-rhamnopyranose, and β-lactose units showed higher antifungal activity against caspofungin–monosaccharide conjugates but not the disaccharide ones. The MIC values against *C. albicans Y0109*, *C. albicans SC5314*, *C. parapsilosis 22019*, *C. krusei* 537, *Cryptococcus neoformans* 32609, *Microsporum gypseum* Cmccfmza, and *Trichophyton rubrum Cmccftla* ranged from 1 to 32 µm/L [64].

Antifungal cyclic and helical-stabilized analogues’ peptide Cm-p5 was synthesized and reported to be active against *C. albicans* and *C. parapsilosis*. Moreover, other derivatives showed antifungal activity against *C. auris* biofilms inhibiting 71–97% using concentrations ranging from 10 to 21 µg/mL [65]. 

The natural phenylalanine-rich cycloheptapeptide segetalin C synthesis was conducted through the coupling and cyclization of peptide units L-Phe-L-ala-L-phe-L-pro-OMe and Boc-gly-L-leu-L-his-OH. The synthesized cyclopeptide showed good antifungal activity against *C. albicans* [66].

### 3.6. Combination Antifungal Activity

Antifungal agents exhibit synergistic effects by acting through various mechanisms to develop their destructive effect against pathogenic fungi. Combination therapy between antifungal drugs has become necessary due to the emergence of antifungal resistance, enhancing their effectiveness against pathogenic fungi that are resistant to single drugs. In their study, Pesee et al. [67] investigated the combination of the lipopeptide antifungal drug caspofungin (CAS) and fluconazole. In vitro experiments revealed a notable influence on the biofilm biomass, cultivable viability, and microstructure of mixed biofilms composed of *C. albicans* and *Candida glabrata*.

To develop effective and specific antifungal compounds, Rodríguez López et al. [68] conducted a study in which they designed amphiphilic, cationic, 14-helical β-peptides. These β-peptides were created to emulate the structural features of natural antimicrobial α-peptides and act as antifungal agents against planktonic *C. albicans* cells. The researchers combined these β-peptides with isoamyl alcohol, which inhibits hyphal formation, resulting in reduced minimum biofilm prevention concentrations (MBPCs) of the β-peptides. These findings suggest that combining peptides from different sources may be a promising approach for combating antifungal resistance in pathogenic fungi.

## 4. Future Perspectives

The rising prevalence of fungal infections, particularly among immunocompromised individuals, has created a need for effective and safe antifungal treatments [69]. In this context, the utilization of cyclic peptides as antifungal agents holds significant promise, and there is a growing interest in exploring their therapeutic applications. Cyclic peptides offer several advantages over conventional antifungal agents, including high potency, low toxicity, and target specificity, as shown by caspofungin, micafungin, and anidulafungin [19,20,23], and the FDA recently approved Rezafungin [70] (a novel echinocandin antifungal agent against invasive *candidiasis* and *candidemia*). Given the success of these drugs, further exploration of other cyclic antifungal peptides is warranted. 

Cyclic peptides, which exhibit diverse antifungal activities, can be obtained from diverse origins, including plants, fungi, and bacteria, and can also be synthesized. Cyanobacteria, for example, were found to produce a wide range of biologically active cyclic peptides that have potential applications in biotechnology and therapeutics [18,19,20,21,22]. The outlook for the isolation of cyclic peptides from fungi is also encouraging. Fungal cyclic peptides have high potential as sources of novel antifungal agents [30,31,32,33,34], and ongoing research is expected to unveil new compounds with different mechanisms of action. Additionally, marine fungi were identified as rich sources of cyclic peptides with antifungal properties.

Future research in this field could focus on identifying new strains of cyanobacteria and fungi that produce unique cyclic peptides with novel structures and biological activities. With advancements in biotechnology and synthetic methods, it is highly likely that more cyclic peptides with potent antifungal activity will be discovered and developed. This could be accomplished by using genomic and transcriptomic techniques to identify biosynthetic gene clusters responsible for cyclic peptide production as well as by employing high-throughput assays to screen for new bioactive compounds [71,72]. Moreover, petidomics is an emerging discipline that integrates genomics, proteomics, analytical chemistry techniques such as liquid chromatography and mass spectrometry, and computational biology to qualitatively and quantitatively analyze biologically significant cyclic peptides [73]. 

Furthermore, the utilization of machine learning algorithms in artificial intelligence (AI) [74,75] enables the extraction of valuable insights and patterns from existing data on the characteristics and functions of acknowledged cyclic peptides. This ability streamlines the search for novel cyclic peptides with specific antifungal properties, derived from both natural and synthetic sources. Moreover, AI aids in accurately predicting the structural properties, including stability and folding patterns, of cyclic peptides. This guidance assists researchers in identifying the most promising candidates for further examination and analysis.

Moreover, the enhancement of existing cyclic peptides derived from cyanobacteria, fungi, and plants via semi-synthetic approaches holds promise for establishing the structure–activity relationship and the creation of more potent and selective analogs with improved pharmacological characteristics such as appropriate pharmacokinetics properties and stability [76,77]. Studying the relationship between the structure and bioactivity of synthesized compounds can provide insights into how different structural features affect the antifungal activity of these compounds. The further investigation of these semi-synthesized compounds as antifungal agents could be pursued through in vivo studies, while advanced biochemical and biophysical methodologies could be employed to unravel their mechanisms of action. Furthermore, these compounds could serve as scaffolds for the development of new drug candidates, optimizing them through medicinal chemistry techniques. Overall, the optimization of cyclic peptides extracted from different sources presents tremendous potential for the identification of novel therapeutic agents and the advancement of biotechnological applications in the coming years.

In the face of antifungal-resistant pathogens [78], the importance of developing combination antifungal therapy has escalated. With the emergence of new strains of resistant fungi, it is crucial to persist in investigating and developing fresh drug combinations that effectively combat these pathogens, thereby reducing the likelihood of resistance development [79]. This approach holds particular significance in the treatment of chronic or recurrent fungal infections, where the emergence of resistance is a notable concern [80]. One avenue for advancing combination antifungal therapy involves merging natural products and synthetic drugs [81]. For instance, researchers can explore the potential of synergizing plant extracts or essential oils with synthetic antifungal agents to create more potent treatment options. Another area of research focuses on combination therapies that target multiple mechanisms of action. By combining antifungal agents with distinct modes of action, researchers can heighten the probability of pathogen eradication while minimizing the risk of resistance development.

The combination of peptides derived from natural, microbial, and synthetic pathways with other drugs displays promising potential in the development of exceptionally effective antifungal agents [82,83,84]. In the future, personalized combination antifungal therapy could be explored. By analyzing specific fungal strains and patient characteristics, researchers may develop tailored combination therapies that are more effective and have fewer side effects.

In summary, cyclic peptides exhibit considerable potential as effective and safe antifungal agents, characterized by their high potency, low toxicity, and specificity. The clinical approval of Caspofungin, Micafungin, and Anidulafungin emphasizes the promising prospects of cyclic peptides in antifungal therapy. However, further investigation is needed to fully exploit the potential of cyclic peptides derived from diverse sources, including cyanobacteria, bacteria, fungi, and plants. Ongoing research and development in this field hold the promise of discovering new cyclic peptides from various origins, significantly broadening the range of available antifungal treatments. Computational methods can aid in the identification and optimization of cyclic peptides with improved antifungal properties. These novel cyclic peptides offer innovative therapeutic options for combating fungal infections in both immunocompromised and healthy individuals, thereby addressing the challenge posed by the widespread prevalence of antifungal-resistant pathogenic fungi. Through continued exploration and advancement, cyclic peptides have the potential to revolutionize the treatment of fungal infections, whether used as standalone therapies or in combination with other drugs, thus contributing to the advancement of global health.

## Data Availability

Not applicable.
